# Development of Drug-in-Adhesive Patch Formulation for Transdermal Delivery of Pelubiprofen

**DOI:** 10.3390/pharmaceutics17121580

**Published:** 2025-12-08

**Authors:** Min-Sung Lee, Chang-Soo Han, Kyung Hyun Min, Dong-Wook Kim, Chun-Woong Park, Kwon-Yeon Weon, Ji-Hyun Kang

**Affiliations:** 1 School of Pharmacy, Jeonbuk National University, Jeonju 54928, Republic of Korea; qyea7172@gmail.com (M.-S.L.); khmin1492@jbnu.ac.kr (K.H.M.); 2 College of Pharmacy, Chungbuk National University, Cheongju 28160, Republic of Korea; hsoo0805@naver.com (C.-S.H.); cwpark@cbnu.ac.kr (C.-W.P.); 3 Respiratory Drug Development Research Institute, Jeonbuk National University, Jeonju 54928, Republic of Korea; 4 Institute of New Drug Development, Jeonbuk National University, Jeonju 54928, Republic of Korea; 5 College of Pharmacy, Wonkwang University, Iksan 54538, Republic of Korea; pharmengin1@wku.ac.kr; 6 College of Pharmacy, Catholic University of Daegu, Gyeongsan 38430, Republic of Korea

**Keywords:** drug-in-adhesive patch, pelubiprofen, oleic acid, acrylic adhesive, silicone adhesive

## Abstract

**Background:** Pelubiprofen (PBF) is a cyclooxygenase-2 inhibitor currently marketed as an oral tablet in South Korea. Oral dosing is limited by gastrointestinal variability, first-pass metabolism, which can reduce therapeutic efficiency and increase adverse effects. Transdermal drug-in-adhesive patches provide a noninvasive alternative that bypasses these limitations and enables controlled delivery through the skin. **Methods:** The solubility of PBF in ethanol was evaluated, and its adhesive compatibility was tested using acrylic- and silicone-based systems. Different drug-loaded formulations were prepared, and their miscibility was assessed. Several permeation enhancers were screened. The physicochemical properties were analyzed. In vitro permeation was studied using rat skin in Franz cells. Accelerated stability was tested at 40 °C and 75% relative humidity for three months. **Results:** PBF reached near saturation at 120 mg/mL in ethanol. Among the adhesives, Duro-Tak^®^ 8076 showed the best compatibility with ethanol and PBF. Drug loading above 15% led to crystallization; 15% was selected as the optimal loading. The addition of 2% oleic acid (OA) significantly increased the permeation flux to 11.31 ± 1.50 μg/cm^2^/h, showing a 3.6-fold enhancement over the control and enhanced deposition in the stratum corneum and dermis. Based on the physicochemical evaluation, PBF was present in an amorphous state within the adhesive matrix. Stability studies revealed no recrystallization, with the drug content maintained at 97–100%. Permeation remained unchanged during storage. **Conclusions:** The PD-OA2 patch achieved stable drug incorporation, enhanced skin permeation, and robust stability. These findings support the potential of PBF as a clinically relevant alternative to oral PBF formulations for treating localized inflammation and pain.

## 1. Introduction

Pelubiprofen (PBF) is a 2-arylpropionic acid derivative and a selective cyclooxygenase-2 (COX-2) inhibitor with a lower incidence of gastrointestinal toxicity than traditional nonsteroidal anti-inflammatory drugs (NSAIDs) [1,2]. By preferentially inhibiting COX-2–mediated prostaglandin E2 production, PBF exerts anti-inflammatory, analgesic, and antipyretic effects [3]. Currently, PBF is marketed only as an oral tablet in South Korea, and no alternative dosage forms have been commercialized. Oral administration is subject to variability in gastrointestinal absorption, hepatic first-pass metabolism, and systemic distribution before reaching the inflamed joints [4,5]. These pharmacokinetic drawbacks may compromise the local therapeutic efficiency and increase the risk of dose-related adverse effects [6].

Transdermal drug delivery systems (TDDS) have emerged as an attractive strategy for overcoming these limitations by enabling controlled drug delivery through the skin [7]. Similar to oral administration, TDDS are non-invasive and suitable for self-administration, as they bypass gastrointestinal metabolism and hepatic first-pass degradation, thereby improving systemic bioavailability [8,9]. TDDS can also provide stable plasma concentrations, minimizing the peak–trough fluctuations commonly observed with oral dosing [10,11]. This is particularly advantageous in the management of chronic diseases that require long-term therapy, as it enhances patient adherence [12,13]. Various transdermal patches have already demonstrated clinical success, including nicotine for smoking cessation, fentanyl for chronic pain, nitroglycerin for angina, estradiol for hormone replacement therapy, and NSAIDs for osteoarthritis and pain [14,15].

In the case of NSAID patches, systemic absorption occurs after application; however, gastrointestinal side effects are generally lower than those associated with oral formulations [16,17]. NSAID patches are widely used for the localized treatment of knee osteoarthritis and pain [18]. Commercially available patches containing ketoprofen or diclofenac have been developed to relieve local inflammation and pain due to their relatively high skin permeability. Nevertheless, the clinical use of these patches is often limited by photosensitivity reactions. Ketoprofen contains a benzophenone-like chromophore capable of absorbing UV light and generating reactive intermediates, which can trigger phototoxic or photoallergic responses. Diclofenac may also produce reactive photoderivatives upon UV exposure, contributing to photosensitivity concerns in patients using diclofenac-based topical formulations [19,20,21,22]. In contrast, pelubiprofen possesses a chemical structure that lacks UV-absorbing chromophores and photoreactive functional groups. This structural advantage markedly reduces the likelihood of phototoxic or photoallergic reactions and also contributes to greater formulation stability under light exposure. These characteristics indicate that pelubiprofen is a suitable candidate for transdermal delivery and may provide a safer alternative to existing NSAID patches with regard to photosensitivity.

Among the TDDS technologies, drug-in-adhesive (DIA) patches have a simple and efficient design [23,24]. In DIA patches, the drug is directly incorporated into a pressure-sensitive adhesive (PSA) layer that adheres to the skin, eliminating the need for separate reservoirs or rate-controlling membranes [25,26]. This thin, flexible design ensures good conformability to curved or mobile body areas and provides reliable adhesion during daily activities [27]. DIA systems also offer significant manufacturing advantages, as drug incorporation and optional permeation enhancers can be achieved in a single blending step, simplifying production and reducing cost [28,29]. Furthermore, their single-layer structure enhances batch-to-batch reproducibility and facilitates large-scale manufacturing [30].

In this study, we aimed to develop and optimize a PBF-loaded DIA transdermal patch for application in localized pain management. Physicochemical characteristics, in vitro skin permeation, drug deposition, and accelerated stability of the optimized formulation were systematically evaluated to confirm its feasibility.

## 2. Materials and Methods

### 2.1. Materials

Pelubiprofen (purity ≥ 99%) was obtained from Daewon Pharmaceutical Co., Ltd., Seoul, Republic of Korea. The acrylic-based adhesives Duro-Tak^®^ 87-9301 and Duro-Tak^®^ 8076 were generously supplied by Henkel (Holthausen, Germany), while the silicone-based adhesives Liveo™ BIO-PSA 7-4302 and Liveo™ BIO-PSA 7-4202 were kindly supplied by Dow (Midland, MI, USA). A casting knife was obtained from LK LABKOREA (Suwon, Republic of Korea). Ethyl alcohol, polyethylene glycol (PG), oleic acid (OA), isopropyl myristate (IPM), transcutol (TC), medium-Chain Triglyceride oil (MCT), and Span 80 (SP) were purchased from Daejung (Seoul, Republic of Korea). All the other chemicals and solvents used were of analytical grade. All experiments were performed using Milli-Q distilled water (Merck, Kenilworth, NJ, USA).

### 2.2. Preformulation Study of a Drug-in-Adhesive Pelubiprofen Patch

#### 2.2.1. Solubility Study

The solubility of PBF in ethanol was evaluated at different concentrations (80%, 90%, and 100% *v*/*v*). Briefly, 200 mg of PBF was added to 1.5 mL of each ethanol solution in a microcentrifuge tube. The mixtures were vortexed for 5 min and then equilibrated at 25 °C for predetermined time intervals (0.25, 0.5, 1, 2, and 24 h). The 24 h time point was included to confirm whether equilibrium had been reached after 2 h. All experiments were conducted in triplicate. At each time point, the suspensions were centrifuged at 13,500 rpm for 5 min, and 0.1 mL of the supernatant was withdrawn and diluted to 100 mL using the mobile phase in a volumetric flask. The diluted samples were filtered through a 0.45 µm polyvinylidene fluoride (PVDF) syringe filter and transferred into vials. The quantity of PBF was measured using high-performance liquid chromatography (HPLC). Analysis was performed using the HPLC system (Vanquish HPLC system, Thermo Scientific, Waltham, MA, USA) and the Aegispak C18 column (150 mm × 4.6 mm, 5 μm) from YoungJin Biochrom (Seongnam, Republic of Korea). The mobile phase consisted of methanol:DW:acetic acid 1200:800:1 (*v*/*v*/*v*) and was eluted at a flow rate of 1.0 mL/min [31]. The detection wavelength was set at 274 nm, and the injection volume was 20 μL. The calibration curve was linear in the range of 7.81 to 250 μg/mL (r^2^ = 0.99995).

#### 2.2.2. Evaluation of Adhesive Compatibility with Ethanol and PBF

The acrylic-based adhesives (DT-8076 and DT-9031) and the silicone-based adhesives (Liveo™ BIO-PSA 7-4302 and Liveo™ BIO-PSA 7-4202) were evaluated by adding ethanol at ratios ranging from 0.1 to 10 (*w*/*w*), relative to an adhesive weight of 1. The mixtures were blended for 30 min and examined for phase separation and precipitation.

PBF was incorporated into a selected acrylic-based adhesive (DT-8076). An ethanolic solution of PBF (120 mg/mL, with solubility determined as described in Section 2.2.1) was prepared and incorporated into the adhesive at PBF contents of 5, 15, 25, 35, and 45% (*w*/*w*, based on the solid content of PBF and the adhesive), as shown in Table 1. The mixtures were blended for 30 min, cast onto a linear film using a casting knife to a uniform thickness of 1.0 mm, and dried in a convection oven at 40 °C for 24 h. Thereafter, the patches were examined for any signs of precipitation.

### 2.3. Formulation of a Drug-in-Adhesive Pelubiprofen Patch with Permeation Enhancers

Permeation enhancers have been screened to improve the transdermal absorption of PBF. As shown in Table 2, PG, TC, IPM, SP, MCT, and OA were evaluated. The concentration of the PBF ethanolic solution (120 mg/mL) was fixed at 15%, and the proportion of the adhesive was adjusted according to the type and amount of enhancer incorporated. The mixtures were blended for 30 min, cast onto a linear film using a casting knife to a uniform thickness of 1.0 mm, and dried in a convection oven at 40 °C for 24 h to obtain the patches.

### 2.4. Physicochemical Characterization

#### 2.4.1. Differential Scanning Calorimetry

Differential scanning calorimetry (DSC) analysis was performed to evaluate the thermal behavior of the formulated patches from the PBF patch samples (approximately 10 mg). The PBF patch samples were analyzed using a DSC 2500 (TA Instruments, New Castle, DE, USA) installed at the Center for University-wide Research Facilities at Jeonbuk National University. Samples were scanned from 0 to 150 °C at a constant heating rate of 10 °C/min.

#### 2.4.2. Fourier Transform Infrared Spectroscopy

Fourier transform infrared spectroscopy (FTIR) was used to investigate the potential interactions between PBF and the excipients. The analysis was performed using a Spectrum Two F-IR Spectrometer (PerkinElmer, Shelton, CT, USA) over a wavenumber range of 4000–400 cm^−1^.

#### 2.4.3. X-Ray Diffraction

X-ray diffraction (XRD) was performed to examine the crystalline properties of the drugs and their formulations. Measurements were performed using a high-resolution XRD (D8 ADVANCE, Bruker, Billerica, MA, USA) over a 2θ range of 5° to 90°. Diffractograms were analyzed to identify the crystalline peaks and assess the physical state of PBF in the patch.

### 2.5. Ex Vivo Skin Permeation Studies

All animal experiments were conducted in accordance with the “Principles of Laboratory Animal Care” and approved by the Committee for the Institutional Animal Care and Use Committee of the Jeonbuk National University. Male Sprague Dawley (SD) rats, 8 weeks old (Samtako, Osan, Republic of Korea), were used for all experiments. The dorsal skin was shaved using an electric clipper, excised, and the subcutaneous fat was removed. The isolated skin samples were stored at −20 °C until use. Prior to the experiment, the skin was thawed at 4 °C for 24 h and then hydrated in 0.1 M phosphate-buffered saline (PBS, pH 7.4) at 37 °C for 3 h before use in Franz diffusion cell studies. The skin permeation studies were performed using a Franz diffusion cell system (Lab FINE, Gunpo-si, Republic of Korea). Rat skin with an average thickness of 1.18 ± 0.11 mm (*n* = 30) was used and cut into 2 × 2 cm pieces prior to loading. Each skin sample was mounted onto a Franz diffusion cell with an effective diffusion area of 1.31 cm^2^. The prepared formulation patches were trimmed to 1 cm^2^ and applied to the skin surface for the permeation experiment. The external temperature of the water bath was maintained at 37 °C, and the skin surface temperature was kept at 32 °C. The receptor chamber was filled with 12 mL of 0.1 M PBS, and SD rat skin was used as the membrane. Permeation studies were conducted at 9, 12, and 24 h. At each predetermined time point, 200 µL of the receptor medium was withdrawn for analysis, and an equal volume of fresh PBS was immediately added to maintain a constant receptor volume. After the diffusion study, drug deposition in the skin was analyzed using the tape-stripping method. The stratum corneum was removed using a 3 M adhesive tape; the first three strips were discarded, and the 4th to 23rd strips were collected for analysis. Each strip was cut into small pieces, immersed in ethanol, and sonicated for 30 min to extract the drugs. The extract was diluted 10-fold with the mobile phase and subsequently filtered through a 0.45 μm PVDF membrane filter before being subjected to validated HPLC-UV analysis.

### 2.6. Accelerated Stability

Accelerated stability testing was performed under controlled conditions of 40 °C and 75% relative humidity (RH) for a period of three months. During storage, potential changes in the drug content, crystallinity of PBF, adhesive properties of the patches, and skin permeation were evaluated. For assay determination, the dried patch was cut into 1 × 1 cm^2^ pieces and extracted in 100 mL of ethanol using a sonicator for 30 min. Subsequently, 1 mL of the extract was withdrawn, diluted 10-fold with the mobile phase, and filtered through a 0.45 μm PVDF membrane filter prior to validated HPLC-UV analysis. The surface morphology of the patches was examined using optical microscopy to detect signs of crystallization. Additionally, XRD analysis was conducted to confirm the physical state of PBF within the matrix and to monitor any potential crystalline changes that may occur during storage. The adhesive strength of the patches was evaluated using a texture analyzer, whereas changes in skin permeation were assessed using Franz diffusion cell studies with excised rat skin.

### 2.7. Statistical Analysis

All data were expressed as the mean ± standard deviation (STD, *n* = 3). Statistical analyses were performed using GraphPad Prism 9.0 (GraphPad Software, San Diego, CA, USA). Group differences were evaluated using the Kruskal–Wallis test, with the PD (control) group as the reference. Statistical significance was defined as *p* < 0.05 (*), *p* < 0.01 (**), and *p* < 0.001 (***).

## 3. Results and Discussion

### 3.1. Preformulation of Drug-in-Adhesive Pelubiprofen Patch

**Figure 1:**
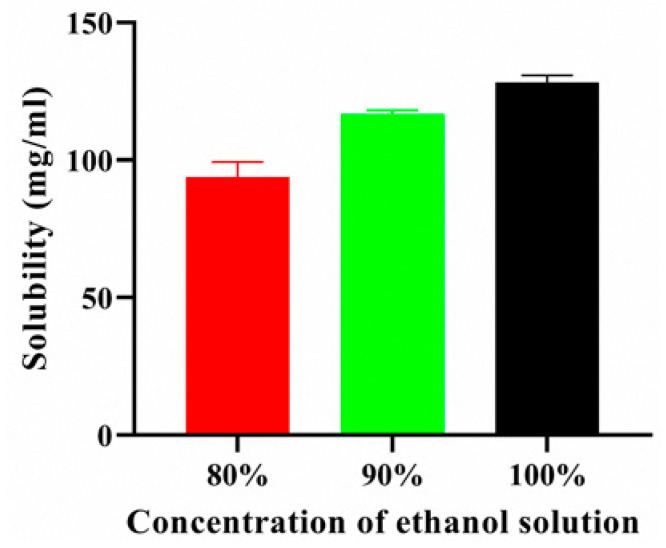
Solubility of PBF in ethyl alcohol.

The solubility of pelubiprofen in ethanol was evaluated at concentrations of 80%, 90%, and 100% (*v*/*v*), and the results are shown in Figure 1. The solubility of PBF increased proportionally with the ethanol content, reaching a maximum of 124.86 mg/mL after 24 h in absolute ethanol (100%). Based on these findings, a working concentration of 120 mg/mL was selected for the subsequent patch formulations. This concentration ensured near-saturated drug loading while avoiding oversaturation, which could lead to precipitation. Therefore, 120 mg/mL provided a practical balance between maximizing drug content and maintaining formulation stability [32].

### 3.2. Adhesive Compatibility with Ethanol and PBF

**Figure 2:**
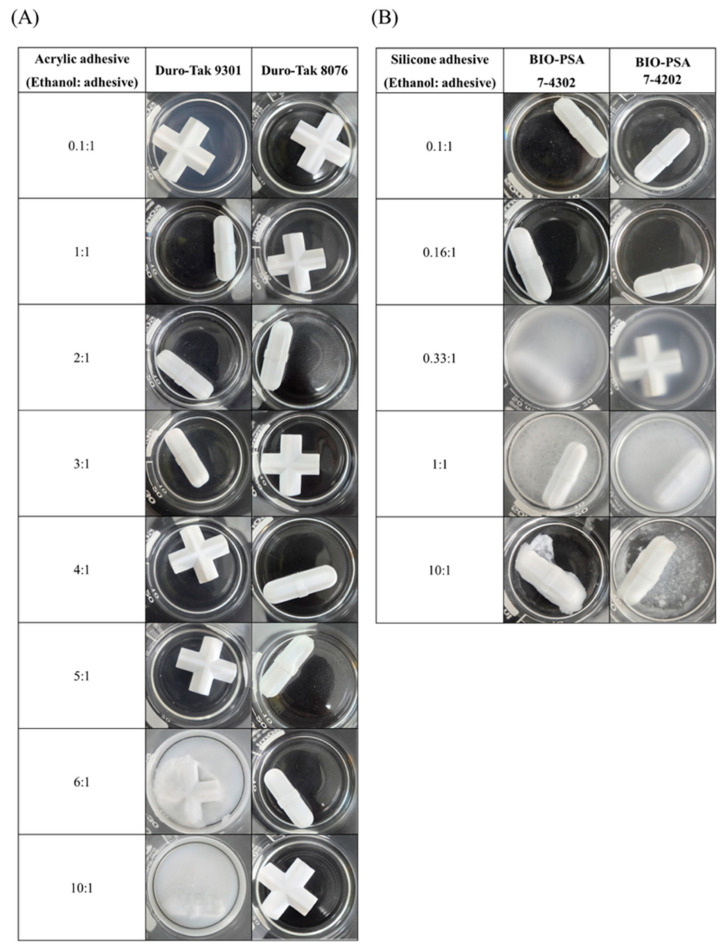
Solvent ratio screening of pressure-sensitive adhesives with ethanol: (**A**) acrylic-based adhesives (Duro-Tak^®^ 9301 and Duro-Tak^®^ 8076) and (**B**) silicone-based adhesives (BIO-PSA™ 7-4302 and BIO-PSA™ 7-4202).

The compatibility of ethanol with various pressure-sensitive adhesives for the solubilization of pelubiprofen was evaluated. The results are presented in Figure 2. For the acrylic-based adhesive Duro-Tak^®^ 9301, a transparent solution was maintained at ethanol-to-adhesive ratios ranging from 1:1 to 6:1. Precipitation was observed at a ratio of 0.1:1. In contrast, phase separation occurred at ratios of 6:1 and 10:1. In contrast, Duro-Tak^®^ 8076 exhibited excellent compatibility, maintaining transparency across all tested ratios (0.1:1 to 10:1). For the silicone-based adhesives BIO-PSA™ 7-4302 and BIO-PSA™ 7-4202, transparent solutions were maintained only at lower ratios (0.1:1 and 0.16:1). Precipitation occurred at higher ratios (0.33:1 and 1:1), and phase separation was observed at a 10:1 ratio. These results indicate that acrylic-based adhesives exhibit broader compatibility with ethanol compared to silicone-based systems, with Duro-Tak^®^ 8076 showing the most favorable performance. The differences in the solvent tolerance can be attributed to the intrinsic properties of the adhesive polymers. Silicone-based PSAs have low polarity and lack strong hydrogen-bonding sites, thereby limiting their miscibility with ethanol. Consequently, excessive ethanol addition leads to supersaturation and precipitation within the silicone matrix, reflecting its poor solvation capacity. In contrast, acrylic-based adhesives inherently contain ester groups (-COOR), which enable partial miscibility with ethanol through dipole–dipole interactions. However, these interactions alone were insufficient to ensure stability at high solvent concentrations. Notably, the presence of stronger polar functionalities, such as -COOH, enabled direct hydrogen bonding with ethanol, thereby significantly enhancing miscibility and stability. Indeed, Duro-Tak^®^ 8076, containing -COOH groups, maintained a homogeneous system at high ethanol concentrations compared with Duro-Tak^®^ 9301, and this is expected to play a crucial role in suppressing premature drug crystallization and preserving uniformity in the drug-in-adhesive system [32,33,34].

**Figure 3:**
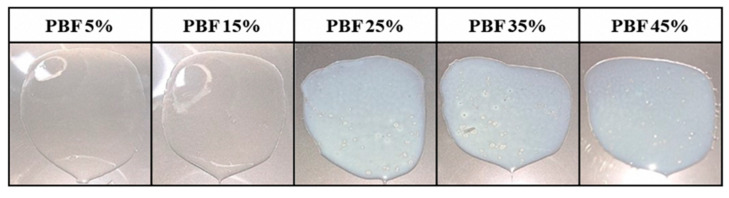
Drug content screening of PBF-loaded patches.

Further investigations were conducted to determine the appropriate drug content. PBF was dissolved in ethanol at a concentration of 120 mg/mL and incorporated into the acrylic-based adhesive Duro-Tak^®^ 8076 at solid mass ratios of 5, 15, 25, 35, and 45% (*w*/*w*). The mixtures were cast and dried to obtain the solid films, as shown in Figure 3. The films containing 25% or more exhibited opacity and visible crystallization upon drying, whereas those containing up to 15% remained clear and homogeneous. The crystallization and opacity observed at loading rates above 25% indicated that the adhesive matrix could not maintain PBF in an amorphous, molecularly dispersed state at higher concentrations. In contrast, patches containing less than 15% of the drug remained transparent, suggesting a uniform distribution and physical stability. Accordingly, a drug loading of 15% was considered optimal, balancing sufficient drug incorporation with the need to prevent phase separation and crystallization, which would compromise formulation performance and reproducibility [35].

### 3.3. Effect of Permeation Enhancers on the Skin Permeation of PBF

**Figure 4:**
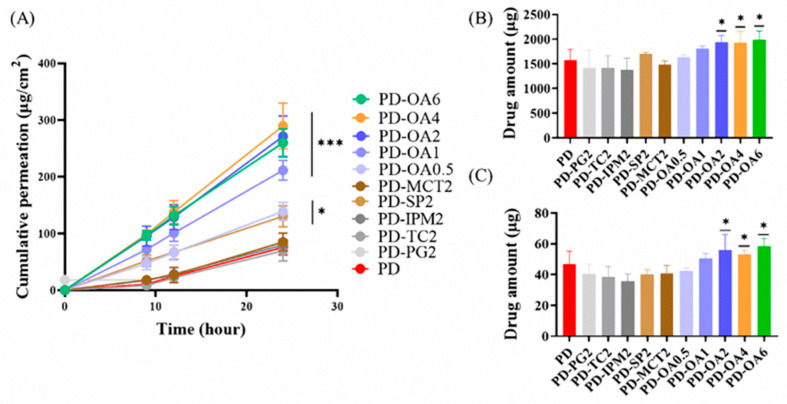
In vitro skin permeation study of PBF patches with permeation enhancer using Franz diffusion cells: (**A**) cumulative permeation profile over 24 h, (**B**) drug amount retained in the stratum corneum after 24 h, and (**C**) drug amount retained in the dermis after 24 h. (*n* = 3) * *p* < 0.05, *** *p* < 0.001 compared with PD.

The effect of penetration enhancers on the PBF-loaded Duro-Tak^®^ 8076 patch formulation (15% *w*/*w*) was investigated, as presented in Figure 4 and Table 3. Drug-in-adhesive (DIA) patches containing 2% of each enhancer were prepared, and their permeation profiles were compared. The rank order of flux values was as follows: PD-OA2 (11.31 ± 1.50 μg/cm^2^/h) > PD-SP2 (5.43 ± 0.78 μg/cm^2^/h) > PD-PG2 (3.55 ± 0.24 μg/cm^2^/h) ≒ PD-MCT2 (3.55 ± 0.66 μg/cm^2^/h) > PD-IPM2 (3.33 ± 0.22 μg/cm^2^/h) > PD (3.16 ± 0.56 μg/cm^2^/h) > PD-TC2 (2.90 ± 0.76 μg/cm^2^/h). Among these, oleic acid (OA, PD-OA2) produced the highest permeation flux, showing a statistically significant improvement compared with the control (PD) (*p* < 0.001). In addition, PD-OA2 resulted in the greatest deposition of PBF in both the stratum corneum (1932.95 ± 146.11 μg) and dermis (55.80 ± 10.28 μg), with statistically significant differences relative to PD (*p* < 0.05). Based on these results, OA was selected as the optimal penetration enhancer, and its concentration-dependent effects were further examined.

Formulations containing 0.5, 1, 2, 4, and 6% OA were evaluated. Compared with PD-OA0.5 and PD-OA1, PD-OA2 showed marked increases in both permeation flux and drug deposition in the epidermis and dermis. All OA-containing formulations demonstrated significantly higher permeation flux than the control (PD), with differences of *p* < 0.001. In addition, OA concentrations of 2% or higher resulted in significantly greater amounts of drug deposited in both the epidermis and dermis compared with PD (*p* < 0.05). However, further increases to 4% and 6% (PD-OA4, PD-OA6) did not yield additional improvements, with values comparable to those of PD-OA2. Mechanistically, OA intercalates into the stratum corneum lipids, disrupting their highly ordered lamellar packing and increasing lipid fluidity, which creates diffusion pathways for drug molecules. This lipid-disordering effect likely accounted for the enhanced penetration and deposition observed in this study. Interestingly, the concentration–response evaluation revealed that the enhancement effect of OA reached a plateau at a concentration of 2%. The significant increases in flux and deposition observed in PD-OA2 compared with 0.5% and 1% OA confirmed that a threshold concentration of OA was required to perturb the stratum corneum barrier sufficiently. However, higher concentrations (≥4%) did not result in further improvement. This plateau phenomenon is consistent with previous reports, indicating that once maximal lipid disruption has been achieved, excess OA may not contribute to additional permeability and could instead risk destabilizing the formulation or causing skin irritation. These findings demonstrate that 2% OA represents the optimal concentration for providing reproducible enhancement of drug permeation while maintaining formulation stability and minimizing the risk of irritation. However, further experimental studies are needed to confirm whether this formulation induces skin irritation, and additional pharmacokinetic evaluations are required to assess its in vivo performance. Moreover, because rat skin typically exhibits higher permeability than human skin, future investigations should also include permeability and safety assessments using human skin to better predict clinical applicability.

### 3.4. Physicochemical Properties of the PD-OA2 Patch

**Figure 5:**
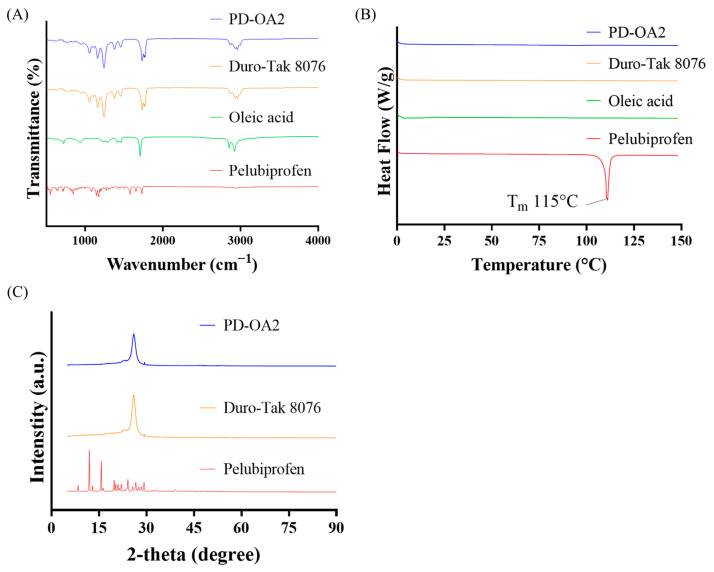
Physicochemical properties of the PD-OA2 patch: (**A**) FTIR spectra, (**B**) DSC thermograms, and (**C**) XRD patterns.

The physicochemical characteristics of the PD-OA2 patches are shown in Figure 5. FTIR analysis of the PD-OA2 formulation showed absorption bands similar to those of Duro-Tak^®^ 8076, with characteristic peaks observed around 1200 cm^−1^ (C–O stretching), 1730 cm^−1^ (C=O stretching), and a broad -COOH band near 2800 cm^−1^. DSC thermograms displayed a sharp melting peak of pure PBF at 115 °C, which disappeared in the PD-OA2 formulation, indicating that PBF existed in an amorphous state. XRD patterns further supported this finding, showing sharp crystalline peaks for pure PBF at 2-theta values of approximately 12° and 16°, whereas only a broad halo was observed for PD-OA2, confirming that PBF existed in an amorphous state within the adhesive matrix. The FTIR spectra suggested that the adhesive matrix primarily governed the spectral features, with no distinct new peaks, indicating chemical interactions between PBF, OA, and the adhesive. Nevertheless, the disappearance of the melting endotherm in the DSC and the loss of crystalline peaks in the XRD clearly demonstrated that PBF existed in an amorphous state within the matrix. Although hydrogen bonding was expected, the FTIR signals were dominated by the adhesive, possibly masking subtle spectral changes. This amorphous dispersion is advantageous because it improves drug miscibility and may enhance skin permeation. The absence of distinct chemical interactions suggests that the structural stability of the adhesive matrix is preserved [36].

### 3.5. Accelerated Stability of the PD-OA2 Patch

**Figure 6:**
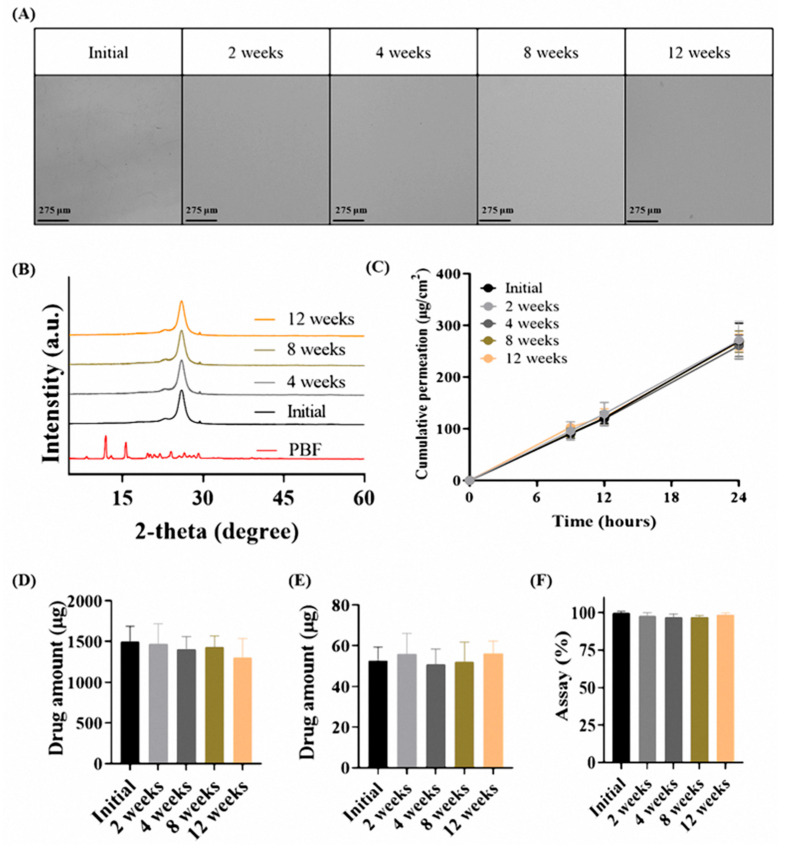
Accelerated stability evaluation of the PD-OA2 patch (40 °C/75% RH, 3 months): (**A**) optical microscopy, (**B**) XRD patterns, (**C**) cumulative permeation profile over 24 h, (**D**) drug amount retained in the stratum corneum after 24 h, (**E**) drug amount retained in the dermis after 24 h and (**F**) assay of patch (*n* = 3).

Stability of the PD-OA2 patch under accelerated conditions was evaluated at 40 °C and 75% RH for three months, and the results are presented in Figure 6. Optical microscopy revealed no recrystallization in the patch, and the morphology remained unchanged compared to the initial state, as shown in Figure 6A. Consistently, XRD analysis showed no crystalline peaks corresponding to PBF at 2-theta values of 12° and 16° after accelerated storage, and the halo peak observed in the initial formulation was maintained, confirming the persistence of the amorphous state.

Franz diffusion cell studies using rat skin further demonstrated that the Q24 values of the 12-week accelerated samples (269.52 ± 34.56 μg/cm^2^) were comparable to those of the initial formulation (267.62 ± 15.21 μg/cm^2^). Similarly, the amounts of drug retained in the stratum corneum (initial: 1500.65 ± 186.14 μg; 12 weeks: 1301.01 ± 236.14 μg) and dermis (initial: 52.62 ± 6.72 μg; 12 weeks: 56.06 ± 6.27 μg) also showed no meaningful differences. No statistically significant differences were observed between the initial and 12-week samples in any Franz cell evaluation parameter. In addition, the drug content remained within 97–100% of the initial value (initial: 100.0 ± 1.01%; 12 weeks: 98.64 ± 1.23%), indicating no significant degradation during storage.

These results verify that the physicochemical integrity of the PD-OA2 patch was preserved under accelerated stress, thus confirming the long-term stability of the formulation. The absence of recrystallization in XRD and microscopy indicates that PBF remained in a stable amorphous form within the adhesive matrix, whereas the consistent flux and drug content supported the preservation of both the physical structure and functional performance. Collectively, these findings demonstrate that the PD-OA2 patch exhibits robust stability under accelerated conditions, highlighting its feasibility for long-term clinical use [37].

## 4. Conclusions

A DIA transdermal patch containing PBF was developed through the systematic optimization of the adhesive type and ethanol-based solvent composition. The optimized formulation (PD-OA2) exhibited adequate drug miscibility and physical stability and significantly enhanced transdermal flux and skin deposition, particularly when oleic acid was incorporated as a permeation enhancer. These findings demonstrate that an appropriate balance among adhesive, solvent, and enhancer composition is critical for maximizing transdermal absorption in DIA patch systems, supporting the feasibility of a PBF transdermal patch that may improve local therapeutic efficacy while overcoming the limitations associated with conventional oral administration. A limitation of this study is that potential skin irritation arising from the drug, adhesive, or permeation enhancer was not evaluated. Additionally, in vivo pharmacokinetic properties of the formulation were not assessed. Future studies should address these aspects to verify systemic exposure, therapeutic relevance, and dermatological safety.

## Figures and Tables

**Table 1:** Composition for PBF Ratio Screening of DT-8076.

PBF Ratio of Solid Components	PBF Ethanolic Solution		Duro-Tak 8076 (Solid Component)
	PBF	Ethanol	
5%	50 mg	0.42 mL	1900 mg (950 mg)
15%	150 mg	1.25 mL	1700 mg (850 mg)
25%	250 mg	2.10 mL	1500 mg (750 mg)
35%	350 mg	2.92 mL	1300 mg (650 mg)
45%	450 mg	3.75 mL	1100 mg (550 mg)

**Table 2:** Formulation composition as the ratio of solid components (*w*/*w*) in the pelubiprofen patch containing permeation enhancers.

Formulation	PBF	Adhesive	Enhancer
PD	15%	85%	-
PD-PG2	15%	83%	2% PG
PD-TC2	15%	83%	2% Transcutol
PD-IPM2	15%	83%	2% IPM
PD-SP2	15%	83%	2% SPAN 80
PD-MCT2	15%	83%	2% MCT oil
PD-OA0.5	15%	86.5%	0.5% OA
PD-OA1	15%	86%	1% OA
PD-OA2	15%	83%	2% OA
PD-OA4	15%	81%	4% OA
PD-OA6	15%	79%	6% OA

**Table 3:** Physicochemical properties and permeation parameters of PBF patches with permeation enhancer (*n* = 3).

Formulation	Drug Content (mg/cm^2^)	Thickness (μm)	Q24 (μg/cm^2^)	Flux (μg/cm^2^/h)
PD	2.90 ± 0.13	211.33 ± 5.25	76.99 ± 9.54	3.16 ± 0.56
PD-PG2	2.65 ± 0.24	217.33 ± 12.47	85.02 ± 11.92	3.55 ± 0.24
PD-TC2	2.81 ± 0.38	211.00 ± 7.23	70.51 ± 1.85	2.90 ± 0.76
PD-IPM2	2.90 ± 0.26	229.00 ± 4.08	70.21 ± 13.02	3.33 ± 0.22
PD-SP2	2.80 ± 0.18	288.00 ± 5.89	131.83 ± 26.75	5.43 ± 0.78
PD-MCT2	2.84 ± 0.36	293.67 ± 12.66	83.77 ± 18.37	3.55 ± 0.66
PD-OA0.5	2.94 ± 0.18	223.67 ± 4.71	140.90 ± 11.81	5.79 ± 0.66 *
PD-OA1	2.97 ± 0.24	228.67 ± 2.36	211.50 ± 9.12	8.82 ± 0.72 *
PD-OA2	3.02 ± 0.24	219.33 ± 4.11	282.63 ± 11.16	11.31 ± 1.50 **
PD-OA4	3.24 ± 0.39	241.33 ± 6.55	260.09 ± 23.74	10.84 ± 1.03 **
PD-OA6	3.00 ± 0.10	230.67 ± 3.09	289.24 ± 48.34	12.08 ± 1.68 ***

* *p* < 0.05, ** *p* < 0.01, *** *p* < 0.001 compared with PD.

## Data Availability

The original contributions presented in this study are included in the article. Further inquiries can be directed to the corresponding authors.

## Abbreviations

The following abbreviations are used in this manuscript:

PBFI	Pelubiprofen
COX-2	Cyclooxygenase-2
NSAID	Nonsteroidal anti-inflammatory drugs
TDDS	Transdermal drug delivery systems
DIA	Drug-in-adhesive
PSA	Pressure-sensitive adhesive
PG	Polyethylene glycol
OA	Oleic acid
IPM	Isopropyl myristate
MCT	Medium-Chain Triglyceride
PVDF	Polyvinylidene fluoride
HPLC	High-performance liquid chromatography
TC	Transcutol
SP	Span 80
DSC	Differential scanning calorimetry
FT-IR	Fourier transform infrared spectroscopy
XRD	X-ray diffraction
SD	Sprague Dawley
PBS	Phosphate-buffered saline
RH	Relative humidity
STD	Standard deviation
